# PAN’s Labyrinth: Molecular Biology of Kaposi’s Sarcoma-Associated Herpesvirus (KSHV) PAN RNA, a Multifunctional Long Noncoding RNA

**DOI:** 10.3390/v6114212

**Published:** 2014-11-04

**Authors:** Cyprian C. Rossetto, Gregory S. Pari

**Affiliations:** Department of Microbiology & Immunology, University of Nevada, Reno School of Medicine, Reno, NV 89557, USA; E-Mail: gpari@medicine.nevada.edu

**Keywords:** Kaposi’s sarcoma-associated herpesvirus (KSHV), gammaherpesvirus, long noncoding RNA, PAN, histone modification, chromatin, epigenetics, gene expression

## Abstract

Kaposi’s sarcoma-associated herpesvirus (KSHV) is an oncogenic γ-herpesivrus, the causative agent of Kaposi’s sarcoma and body cavity lymphomas. During infection KSHV produces a highly abundant long non-coding polyadenylated RNA that is retained in the nucleus known as PAN RNA. Long noncoding RNAs (lncRNA) are key regulators of gene expression and are known to interact with specific chromatin modification complexes, working in *cis* and *trans* to regulate gene expression. Data strongly supports a model where PAN RNA is a multifunctional regulatory transcript that controls KSHV gene expression by mediating the modification of chromatin by targeting the KSHV repressed genome.

## 1. Introduction

Kaposi’s sarcoma-associated herpesvirus (KSHV) was first identified as the etiological agent of Kaposi’s sarcoma in 1994 [1], and it wasn’t long after that before researchers first identified a highly abundant viral RNA from cells that were infected with KSHV [2,3]. Originally termed T1.1 or nut-1 RNA (U-RNA like nuclear transcript), this highly abundant RNA was identified as a 1.1-kb polyadenylated viral transcript. The coding potential of T1.1 was dismissed based on two factors. First, there are a few possible open reading frames but they are at suboptimal positions within the transcript and would code for very small proteins. The second reason T1.1 was extremely unlikely to have any coding potential was its location within the cell; it was found to be primarily located in the nucleus of infected cells. Cytoplasmic and nuclear fractionation studies of 293T cells transfected with a T1.1 expression plasmid demonstrated that it was only detectable in the nuclear fraction and *in situ* hybridization studies also localized PAN RNA to the nucleus of KSHV infected cells [2]. These same studies also quantitated the relative copy number of T1.1 transcripts to ~25,000 copies per cell based on the signal intensity of the same cells with cellular U12 RNA. During lytic infection PAN RNA is estimated to account for over 80% of the viral transcriptome. This apparent high expression level allows for the possibility that PAN RNA is a major factor with respect to contribution to KSHV replication and growth.

**Figure 1:**
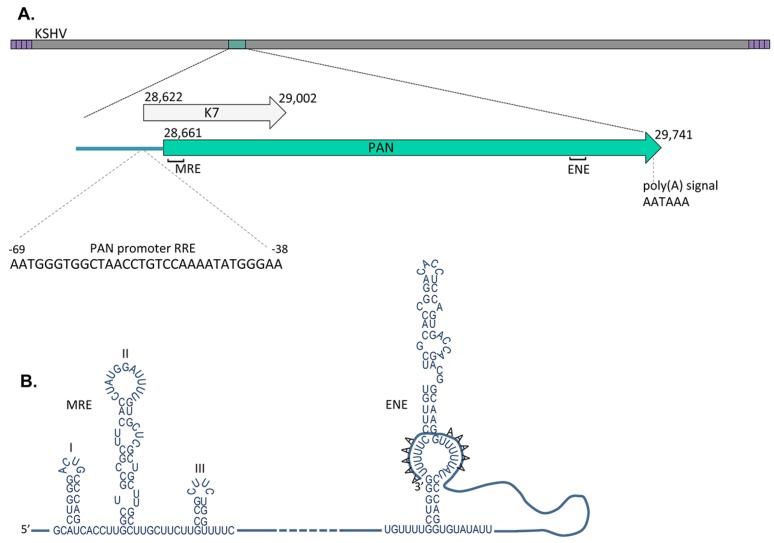
Schematic diagram depicting key features of the PAN locus and transcript. (**A**) The nucleotide numbering for the K7 ORF and PAN transcript corresponds to genbank accession number U75698.1 for HHV8/KSHV genome type M isolated from BC-1 cells. The numbering for the RRE within the PAN promoter and the ENE is from the transcription start site; (**B**) The secondary structure of the ENE and MRE sequence within PAN RNA to prevent degradation and increase stability.

The PAN gene locus is found in the KSHV genome between K6 and ORF16, and slightly overlaps it’s 5' end with the 3' end of the K7 ORF (see Figure 1). PAN RNA corresponds to nucleotides 28,661–29,741 (accession number U75698.1) in the KSHV genome. Molecular analysis revealed a strong K-Rta responsive promoter upstream of the PAN RNA start site, and a poly(A) signal sequence at the 3' to promote RNA stability.

More recent sequencing analysis using next generation sequencing technology has offered further insights into PAN RNA. A 2014 study by Arias *et al.* demonstrated that during lytic induction PAN RNA accounts for 65.5% of the KSHV reads as soon as 8 h post induction (hpi), at 24 hpi PAN accounts for 78.5%–92.2% of KSHV reads, and at 48 hpi PAN accounts for 84.8%–91.9% of KSHV reads [4]. There have also been reports of PAN RNA detected in latently infected, non-induced cells. Detection of PAN RNA during latency has often been attributed to spontaneous lytic reactivation [4]. Work from our laboratory demonstrated that a significant amount of PAN RNA is expressed from a virus mutant defective for expression of the major transactivator K-Rta [5]. Additionally, we showed that we could detect PAN RNA in uninduced BCBL-1 cells and PFA treated BCBL-1 cells. 

There has also been a report of a transcript that is antisense to PAN and K7, termed K7.3, discovered using tiling microarray analysis [6]. Using next generation sequencing techniques along with a strand oriented RNA-seq library preparation kit we were unable to detect any appreciable amounts of the antisense PAN/K7 transcript. Due to differing isolation techniques and treatment of the RNA, it remains to be seen whether this antisense transcript is a true RNA species with viral kinetics that can be classified into a temporal category.

## 2. Long Noncoding RNAs (lncRNAs)

The discovery of lncRNAs has lead to increasing evidence that they play a prominent role in human disease [7]. Many examples now exist where specific lncRNAs can reprogram chromatin and promote disease or changes in gene expression [8,9,10,11,12]. The ability of these novel transcripts to regulate gene expression is an exciting new area for research. Although the field of lncRNAs is still developing, several mechanisms have been postulated as to how lncRNAs can impact gene expression [7,13,14]. There are four main proposed mechanisms of action for lncRNAs, these include acting as a decoy, a scaffold, a guide, or an enhancer. A decoy lncRNA acts as a “sponge” to isolate proteins, such as transcription factors or chromatin modifiers, away from their normal substrates. A scaffold lncRNA is used to assemble protein complexes; usually these types of lncRNAs will have unique domains to bind different proteins creating a complex that is functionally distinct from its individual parts. A guide lncRNA acts to localize specific proteins, often this can be a specific chromatin target to cause changes in gene expression. An enhancer lncRNA facilities the signals from transcription factors to regulate gene expression. Another interesting model of lncRNA’s, which goes along with its mechanisms of action, is that they can regulate nuclear organization to orchestrate and traffic protein complexes, genes, and chromosomes to appropriate locations allowing for proper activation or repression [15].

lncRNAs associate with chromatin modifying complexes [12] and interact with components of the polycomb repressive complex 2 (PRC2). The PRC2 complex is composed of EZH2, SUZ12 and EED-1. EZH2 is a protein that adds three methyl groups to lysine 27 of histone 3 [16]. SUZ12 is a protein that contains a zinc finger domain that is the point of contact with RNA [17]. EED-1 interacts with HDAC1 and histone deacetylase and various other proteins to mediate gene repression [18]. These PRC2 proteins can mediate changes in histone modifications (methylation) and subsequent repression of gene expression from various genetic loci. Hence the interaction of lncRNAs with PRC2 can globally influence gene expression.

Although the ability of lncRNAs to repress gene expression is well documented, more recent reports suggest that they are multifunctional having the ability to activate, as well as suppress gene expression. One interesting example of this is the lncRNA, lincRNA-Cox2, which can both activate and repress gene expression associated with immune response [19].

While thousands of lncRNAs with diverse functions have been identified in mammalian cells, the exact function or mechanisms of action for most of these transcripts are unknown. Several technological advances have been described that can be used to study lncRNAs. The use of techniques, such as Chromatin Isolation by RNA purification (ChIRP), and the more recent domain-specific chromatin isolation by RNA purification (dChIRP), are invaluable with respect to mapping specific lncRNA DNA interaction domains [20,21].

## 3. PAN Expression during Lytic Reactivation and Latency

The expression of PAN RNA during KSHV lytic infection is regulated by major viral transactivator K-Rta through the interaction of a specific DNA interaction domain referred to as Rta-Responsive Element (RRE) [22,23]. The RRE is located between nts −69 and −38 of the PAN RNA promoter and directly interacts with K-Rta to activate transcription [24] (Figure 1A). *In vitro* and *in vivo* studies of the interaction between the PAN promoter and K-Rta protein have demonstrated that K-Rta has an extremely high affinity for the PAN promoter, even under stringent high-salt conditions [22,24]. PAN RNA is stabilized by the interaction of ORF57 [25]. PAN RNA expression is also upregulated and stabilized by an interaction of ORF57 with PABPC1 [26]. Interestingly, in transient assays the viral encoded K-bZIP, which was shown to interact with K-Rta, suppresses transcription from many KSHV promoters has no affect on the PAN RNA promoter [27]. In addition to K-Rta mediated expression of PAN RNA, it was shown that the cellular factor CAAT/enhancer-binding protein alpha (C/EBP) cooperates with K-Rta to activate PAN RNA expression [28]. 

Although K-Rta activates PAN RNA expression upon reactivation, it is clear that a K-Rta independent mechanism also exists. Recombinant viruses defective for K-Rta expression still express PAN RNA, suggesting that PAN RNA is present during all phases of the KSHV life cycle [5]. It should also be noted that although PAN RNA is present at early times post infection, a significant amount of the RNA in packaged virions is PAN RNA [5]. This observation reinforces the concept that PAN RNA is a ubiquitously present regulatory factor in KSHV infected cells. Recent studies show that PAN RNA interacts with the KSHV latency associated nuclear antigen, LANA [29]. Studies show that the *N*‑terminus (as 1–70) of LANA are involved in the interaction with PAN RNA. Also, the 3' end of PAN RNA contained within nucleotides 750 to 1062 was shown to be the critical interaction domains that bind to LANA. This interaction is thought to serve to sequester LANA away from viral DNA episomes to facilitate lytic reactivation.

## 4. *Cis*-Acting Elements, Triple Helix Structure and Localization of PAN RNA

The relative abundance and localization of PAN RNA was speculated to be attributed to specific physical characteristic unique to PAN RNA, further studies would go on to demonstrate that PAN RNA contains at least two *cis*-acting elements that contribute to it’s stability and localization. One of the early insights into the function of PAN RNA comes from the elucidation of an RNA element within PAN RNA that mediates nuclear retention. PAN RNA contains an expression and nuclear retention element (ENE) that allows for formation of a triple helix, such that the Poly(A) tail of PAN RNA is sequestered, retaining the transcript in the nucleus [30,31,32]. There have been many interesting studies that have focused on discerning the structure of PAN RNA that allows for it’s unique stability and nuclear retention.

The ENE 79-nt sequence within PAN RNA was originally described and characterized by Conrad and Steitz [31]. They reasoned that because PAN RNA was expressed at such high levels, and was not spliced or exported to the cytoplasm, they should look for the presence of a *cis*-acting element that could confer those unique characteristics to PAN RNA. They also performed studies to insert the PAN ENE within an intronless β-globin mRNA, and demonstrated that the fusion RNA transcript accumulated to high levels and was retained in the nucleus. Using the β-globin-ENE as a type of reporter RNA, they also demonstrated that tethering of export factors or splicing could overcome the retention signal that the ENE confers to the RNA. One interesting caveat was noted about the ENE, and most likely points to other *cis*-acting elements within PAN RNA, multiple copies of the ENE were required to increase the amount of the β-globin transcript retained in the nucleus and deletion of the ENE within PAN did not cause an increase in PAN RNA’s cytoplasmic accumulation.

The identification of the ENE within the 3' end of PAN RNA eventually lead to physical analysis of PAN RNA’s secondary structure. When the ENE was first discovered there was speculation that the unpaired stretches of U’s in the ENE might hybridize with the poly(A) tail and protect the polyadenylated 3' end from exonucleolytic attack, and this would account for one of the reasons why PAN RNA was so stable [33]. Further investigation revealed that it wasn’t just the amount or number of U’s in the ENE that accounted for the accumulation of PAN RNA, there was a structural stem-loop component of the ENE that added to the stability of PAN RNA. The crystal structure of the ENE core with the poly(A) sequence revealed that the U-rich loop of the ENE sequestered the poly(A) tail by the formation of a major-groove triple helix thereby blocking the initiation of RNA decay [30]. 

Identifying the ENE and triple helix formation within PAN RNA has lead to recognizing similar ENE types of *cis*-acting elements within other lncRNA’s. Using bioinformatics and computation approaches has aided in the identification of ENE *cis*-elements in other viruses such as rhesus rhadinovirus (RRV) and equine herpesvirus 2 (EHV2) [32]. Further studies looking at cellular lncRNA’s have found an ENE and bipartite triple helix within metastasis-associated lung adenocarcinoma transcript 1 (MALAT1) transcript [34,35]. Secondary structure for RNA’s, and lncRNA’s in particular, play an important role in their regulation, stability, and functional targeting. Find additional *cis*-elements like the ENE and triple helix formation will help us better understand the molecular interactions between lncRNA’s and their substrates.

Despite the presence of the ENE, more recent evidence supports stabilization of PAN RNA through a 9-nt element termed the MRE (Mta-responsive element) [25]. ORF57, the viral encoded Mta protein, was identified as a possible factor in modulating PAN RNA metabolism. The relative locations of MRE and ENE are shown in Figure 1B. Interestingly, in the absence of viral infection and other viral encoded factors, PAN RNA is stably expressed in cell lines and interacts with cellular DNA to activate or repress gene expression [5,36]. There have been many reports that support the interaction of PAN RNA with ORF57 [25,26,37,38,39]. Studies have described the ability of ORF57 to prevent the decay of PAN RNA even in a PAN-∆ENE mutant, and identified a larger 300 bp section called the ORF57-responsive element (ORE) that encompasses the smaller MRE [39]. Deletion studies of the ENE and MRE have clearly shown that the ENE plays only a small role in ORF57-mediated PAN stability, while the major function of the MRE is to increase the half-life of PAN RNA by its interaction with ORF57 [25]. This same study also reported that approximately 20% of PAN RNA was exported to the cytoplasm, and by using a plasmid co-transfection system of PAN RNA and ORF57, they were also able to detect 30% of PAN RNA in the cytoplasm when ORF57 was co-expressed along with PAN. While studies that used the larger 300 bp ORE were able to demonstrate that the ORE was able to confer an ORF57 depend accumulation on heterologous intronless mRNA [39]. Insertion of the MRE into other heterologous mRNA, such as vGPCR and luciferase that are normally insensitive to the effects of ORF57, showed only minimal increase in mRNA accumulation [25]. Insertion of the minimal MRE along with the ENE also showed no added increase in mRNA accumulation [25], indicating that there may be other *cis*-elements or motifs within PAN RNA that contribute to its extremely high abundance.

## 5. PAN RNA as a Regulatory Factor

As regulatory RNA, PAN associates with several viral and cellular factors. Early reports described the interaction of PAN RNA with KSHV encoded ORF57 [37,38]. ORF57 increases the stability of PAN RNA and increases its nuclear concentration [25,39]. The interaction of ORF57 is specific and mediated through a core MRE found within the 5'-end of PAN RNA [25,40]. The mechanism involved in ORF57 mediated stability of PAN RNA involves the recruitment of factors composed of the TREX complex [41]. PAN RNA interacts with cellular polyadenylate-binding protein C1 (PABPC1), which binds to PAN RNA through the MRE located in the 5'-end of the PAN RNA transcript and has a negative affect on PAN RNA stability [25,26]. 

Studies involving the elucidation of a mechanism for the increased stability and nuclear localization of PAN RNA were significant. However, none of these early endeavors unlocked the mystery with respect to the function of PAN RNA in infected cells. Proteomics approaches from the Steitz lab and our lab [42,43], using two slightly different procedures to isolated proteins interacting with PAN RNA, provided some insight into the binding partners of PAN RNA. The first studies involving a proposed function for PAN RNA revealed that depletion of PAN RNA using antisense in infected cells impaired KSHV late gene expression and decreased the amount of infectious virus [42]. Subsequent studies showed that PAN RNA also interacted with histones H1 and H2A, mitochondrial and cellular single-stranded binding proteins and interferon regulatory factor 4 (IRF4) and KSHV ORF59 [43]. Transient assays showed that PAN RNA interfered with the ability of IRF4/PU.1 to activate the interleukin-4 (IL-4) promoter. Evaluation of the expression of other cellular immune response genes also showed a decrease in expression of other factors involved in immune response [43]. These studies illuminated the first clues with respect to a possible function for PAN RNA, a modulator of immune and inflammatory response. 

## 6. PAN RNA and Viral Replication

The ability of PAN RNA to disregulate the expression of immune response genes and those involved in formation of the inflammasome suggested that PAN RNA had a global affect on cellular and viral gene expression programs. To further investigate the role of PAN RNA in KSHV growth, we generated a recombinant KSHV BAC with a large deletion in the PAN RNA gene [44]. This viral mutant failed to express most of the viral lytic gene expression program under reactivation conditions. The over-expression of K-Rta (ORF50), the major viral transactivator protein, failed to rescue the observed gene expression defect, suggesting that PAN RNA was required for full activation of KSHV gene expression and virus production [44]. The mechanisms involved in the ability of PAN RNA to activate KSHV and cellular gene expression is directly related to the interaction of PAN RNA with chromatin modifying complexes. PAN RNA binds to UTX and JMJD3, demethylases that specifically removes the methyl groups from the repressive H3K27me3 mark, and MLL2, a histone-lysine *N*-methyltransferase, which methylates H3 at the K4 position, a mark associated with gene activation [44,45,46]. ChIRP-Seq showed that PAN RNA occupies much of the KSHV genome during lytic infection including the PAN RNA gene promoter itself. PAN RNA also interacts with protein components that comprise the polycomb repression complex 2 (PRC2). It was previously demonstrated that during latency the KSHV genome is composed of both activating and repressive histone marks [47]. However, most of the latent genome is associated with repressive H3K27me3 and H3K9me3 repressive marks with the ORF50 promoter and some early gene promoters associated with both repressive and H3K4me3 and acetylated H3 activation marks. It could be postulated that PAN RNA mediates latency or reactivation through its interaction with different chromatin modifying complexes, since PAN RNA is present in packaged virions and is expressed in the absence of K-Rta. Although the PAN RNA deletion mutant does establish latency in HEK293 cells, it is possible that in the appropriate target cells PAN RNA may be required to promote KSHV latency. 

## 7. Targeting of PAN RNA to KSHV Genomic DNA

One of the least known mechanisms for lncRNAs is how these molecules are targeted to specific DNA loci. For KSHV, some insight into this targeting mechanism could lie in the observation that PAN RNA interacts with ORF59 [43]. KSHV ORF59 encodes the polymerase processivity factor [48,49]. Interestingly, the human cytomegalovirus (HCMV) lncRNA4.9 also interacts with the HCMV encoded processivity factor UL44 [50]. Although originally described as a binding partner for herpesvirus-encoded polymerases, processivity factors are emerging as having diverse functions in viral replication and gene expression [51,52,53,54,55,56,57,58]. For KSHV, ORF59 is associated with the same regions of the viral genome as PAN RNA occupancy, strongly suggesting that ORF59 is involved in the targeting mechanism or anchoring of PAN RNA to specific regions of the KSHV chromosome. Hence PAN RNA, like most lncRNAs, has a complex three-dimensional structure that allows for specific protein binding and RNA-DNA hybridization through openly configured single-stranded sequences, which cannot be predicted by the primary RNA sequence. These two characteristics of lncRNAs, protein binding and recognition of specific DNA sequences, imparts functionality.

## 8. PAN RNA and Cellular Gene Expression

A later investigation of the ability of PAN RNA to destabilize cellular gene expression showed that PAN RNA interacts with polycomb group proteins, which are associated with repression of gene expression [5]. Interestingly, PAN RNA was shown to globally disregulate cellular gene expression programs with a concentration on the expression of factors involved in inflammation, immune response and cell cycle. Cells that constitutively express PAN RNA grow to a higher density and have shorter doubling times consistent with an increase in cell survival [5]. This dramatic increase in cell survival mediated by PAN RNA allows for increased viral fitness. PAN RNA expressing cell lines can be efficiently infected with KSHV and are easily transfected with KSHV BACmids, suggesting that PAN RNA promotes a cellular environment highly advantageous to viral growth [59]. 

**Figure 2:**
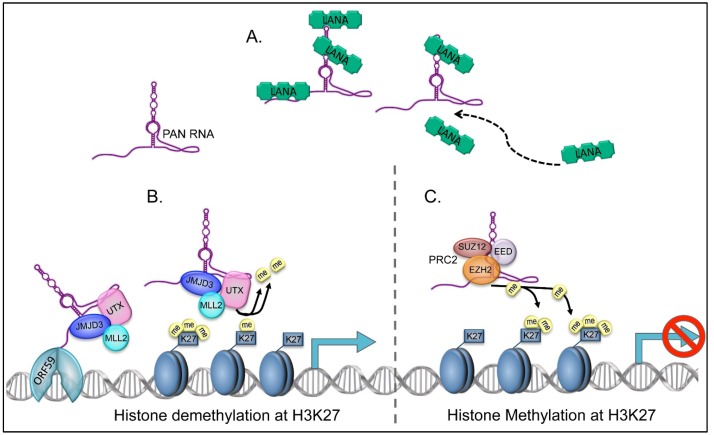
PAN RNA both activates and represses gene expression depending on its interaction with chromatin modifying complexes. (**A**) The interaction of PAN RNA with LANA during reactivation is partly responsible for the LANA-episome dissociation allowing for the expression of lytic transcripts. In this model PAN RNA acts as a “sponge” to keep LANA from repressing viral gene expression; (**B**) The interaction of PAN RNA with UTX/JMJD3 targets H3K37 for demethylation and acts to increase gene expression. The interaction of PAN RNA with viral encoded processivity factor ORF59 may aid in the targeting UTX/JMJD3 to viral genes during lytic gene expression; (**C)** PAN RNA’s interaction with PRC2 targets H3K27 for methylation and acts to repress gene expression. The mechanism for specific targeting of PAN RNA is currently under investigation.

As mentioned previously, in addition to interacting with chromatin modifying complexes that activate gene expression, PAN RNA interacts with factors associated with the PRC2 [5]. Hence, the interaction with PCR2 factors as well as UTX. JMJD3 and MLL2 (KMTD2), a histone methyltransferase, allows for PAN RNA to both activate and repress gene expression (see Figure 2) [44]. The differential association of PAN RNA with specific chromatin modifying complexes may be key to understanding the mechanism of action for PAN RNA during different phases of the KSHV life cycle. Although the precise mechanism involved in the modulation of PAN RNA mediated activation or repression is unknown, ChIRP-Seq results clearly show that PAN RNA interacts with hundreds of cellular promoters for genes that express factors involved in a wide spectrum of host functions.

## 9. Coding Potential of PAN RNA

There has always been interest in the speculation about the coding potential of PAN RNA. In the early studies on PAN RNA, it was originally localized to the nucleus, lacked a 5' trimethylguanosine cap, and did not associated with ribosomes [3]. For these reasons, plus the fact that within PAN RNA there are stretches of sequence similar to cellular non-coding RNA U1, most researchers assumed that PAN RNA was non-coding. Proteomics LC-MS/MS analysis from latent BCBL-1, latent and lytic BC-1 cells did not detect any peptides that correspond to the potential PAN coding regions, however it was acknowledged in the same study that the proteomics is limited and may not fully characterize under-represented proteins [6]. The most recent development in the coding potential of PAN RNA comes from a study that used mRNA-Seq, along with ribosome footprinting (Ribo-Seq) and DNA-Seq, to compile a comprehensive analysis of viral gene expression [4]. The comprehensive analysis not only included a time course including during latency and following lytic reactivation, but also chemical treatment with harringtonine to arrest the progression of initiating ribosomes and cycloheximide (CHX) to arrest elongating ribosomes. Interestingly the PAN RNA followed classical ribosome protection patterns, they found initiating ribosomes accumulating at the start codons in the samples treated with harringtonine, elongating ribosomes in the body of the transcript in CHX treated samples, and releasing ribosomes at the stop codon in the samples that were not treated with an translation inhibitor. In this study the three predominant putative PAN ORFs are termed PAN1.1 (37aa), PAN1.2 (44aa), and PAN1.3 (25aa). The calculations of the ribosome release score (RSS) are comparable to those of known coding RNAs, and the low translation efficiency of the three major PAN ORFs is offset by the significant amount of PAN RNA that accumulates during the lytic cycle. This study calculated the ribosome-protected RNA corresponding to the small PAN peptides represented up to 1.7% of the total CHX Ribo-Seq reads, strongly suggesting that the putative peptide could be quite abundant.

Although the coding potential of PAN RNA is debatable, there is agreement that because PAN RNA is extremely abundant and that there is more than enough RNA to be translated and act as a long non‑coding RNA. Those arguing against the coding potential of PAN would cite the fact that early studies of PAN RNA did not find it associated with a 5' trimethylguanosine cap [3], the 5' trimethylguanosine cap is most often associated with cellular small nuclear RNA’s such as U1 RNA, which was used as the control in the original study. The lack of a 5' cap means that it would most likely not be exported out of the nucleus using the nuclear pore complex and if cytoplasmic localization were achieved, it would have to rely on cap-independent translation such as an internal ribosome entry site (IRES). Later studies have often noted PAN RNA has a 5' cap [30,60], but a search of relevant literature shows a lack of experimental proof that PAN RNA did not associate with a trimethylguanosine cap. Interestingly, one study focusing on developing the function of the ENE, found that the effects of the ENE were similar whether they used GpppG or m^7^GpppG capped substrates but the data was not shown [60]. The exact state of the 5' cap on PAN RNA has not been completely confirmed or disproven, and the ambiguity may lie between not having a 5' trimethylguanosine cap [3] and possibly having a 5' monomethylguanosine cap [60]. Since PAN RNA is transcribed by RNA polymerase II, capping enzymes associated with the RNA pol II after the CTD of RNA pol II has been phosphorylated and during the elongation phase of RNA pol II, should cap the 5' end during the transcription resulting in a 5' monomethylguanosine cap.

It has been shown that PAN RNA is polyadenylated and binds to poly(A)-binding protein C1 (PABPC1) after PABPC1 is relocalized to the nucleus during lytic infection [42]. This is particularly interesting because PABPC1 is normally found in the cytoplasm bound to poly(A) tails of mRNA and regulates mRNA stability and translation. The fact that PABPC1 binds to PAN RNA in the nucleus during a lytic infection could be indicative of the virus manipulating the host environment to favor the export and expression of viral mRNAs. A number of studies using *in situ* hybridization [3,39,42] and subcellular fractionation [2] localized PAN RNA to the nucleus. In BCBL1 cells treated with valproate to induce the virus to enter the lytic cycle, approximately 20% of PAN RNA was exported to the cytoplasm. The fact that most PAN RNA resides in the nucleus and not in the cytoplasm argues against proteins being translated from PAN RNA.

## 10. Conclusions

Since the discovery of PAN RNA as the most abundant viral transcript during a KSHV infection, there has been much effort put forth to uncover how PAN RNA achieves and maintains a very high steady state level of accumulation. Molecular analysis revealed PAN RNA is transcribed from a K-Rta responsive promoter, which binds K-Rta at a higher affinity than other K-Rta responsive promoters, resulting in higher levels of transcription. The other key to PAN RNA’s abundance is conferred by it’s unique secondary structure of the ENE which sequesters the poly(A) tail to prevent degradation and the interaction with ORF57 in infected cells. As work was going forward to discern PAN’s molecular characteristics, at the same time there were theories about what functional benefit PAN RNA may impart to the virus. Our lab and other’s have demonstrated that PAN RNA is essential for viral late gene expression, as shown using a virus lacking the PAN locus and alternatively using antisense oligonucleotides to degrade PAN RNA. PAN RNA can regulate cellular and viral gene expression by targeting chromatin‑modifying complexes for either activation or repression. This fact cannot be overstated since PAN RNA is the most abundant transcript in lytically infected cells and is present in the absence of ORF50 (K-Rta) expression. Therefore PAN RNA has the potential to function as a master regulator of viral gene expression. 

It is now well established that lncRNAs participate in a wide variety of biological processes. New and better techniques and reagents are developed to decipher the mechanism of action for lncRNAs. Nevertheless it is clear that lncRNAs have the capacity to act as decoys, scaffolds, guides, or enhancers. Viral lncRNAs are only very recently being recognized as playing a significant role in regulation of gene expression similar to their cellular counterparts. For viral lncRNAs, their high abundance compared to the expression levels of protein coding mRNAs makes them hard to ignore as modulators of gene expression and chromatin modification.
